# Representation of Autobiographical Memories Along a Sagittal Front-to-Back Mental Timeline: Evidence from Mandarin Speakers

**DOI:** 10.3390/bs16030314

**Published:** 2026-02-25

**Authors:** Ying Sun, Ying Fang, Wenxing Yang

**Affiliations:** College of Foreign Studies, Yangzhou University, Yangzhou 225127, Chinafangy@yzu.edu.cn (Y.F.)

**Keywords:** autobiographical memory, mental timeline, metaphor, Mandarin

## Abstract

Accumulating evidence over the past decades has established that people conceptualize elapsing time along a sagittal mental timeline (MTL). A recent study discovered that representations of autobiographical memories (AMs) also proceed along a sagittal back-to-front MTL, consistent with the direction of sensorimotor experiences such as walking or running. The present investigation attempted to clarify and extend that work by exploring if the back-to-front axis for the temporal organization of AMs is a universal phenomenon across linguistic communities. An experiment that recruited Mandarin speakers as participants was conducted. The experimental task asked participants to categorize personal events retrieved from their AMs as past- or future-related via distinct key arrangements that corresponded to a back-to-front and a front-to-back line respectively. Results show that cross-linguistic variations may exist in the directionalities of MTL underlying AM processes. Contrary to the back-to-front MTL observed among Italian speakers in the aforementioned research, Mandarin speakers conceived of AM progression as oriented from front to back. The findings of the present study provide preliminary evidence to validate the predictive power of spatiotemporal metaphors rather than sensorimotor experience in shaping a sagittal MTL for AM representations, especially when the two forces contradict each other in terms of spatial directions.

## 1. Introduction

Time is an abstract concept and cannot be directly perceived. Around the world, people may think and reason about time in the concrete and tangible domain of space (Boroditsky, 2000; Casasanto & Boroditsky, 2008; Hendricks et al., 2018; Núñez & Sweetser, 2006; Pan & Huang, 2024). It is well-documented in the literature that elapsing time is represented along a hypothetical sequentially oriented line that moves from one extremity to another (Bonato et al., 2012; Santiago et al., 2010; Valenzuela et al., 2020). This continuous linear spatial path is commonly referred to as the mental timeline (MTL). Shaped by various linguistic, cultural and personal elements, the specific dimensions of MTLs can be mapped onto different spatial axes. The most prevalent internal space–time associations, as evidenced by abundant experimental and theoretical works (e.g., Beracci & Fabbri, 2024; Casasanto & Jasmin, 2012; Chrabaszcz et al., 2025; Dalmaso et al., 2023; Fuhrman & Boroditsky, 2010; Gu et al., 2019; Miles et al., 2010; Ouellet et al., 2010; Sun et al., 2022; Walker et al., 2017), take the form of a sagittal (front–back) axis, a horizontal (left–right) axis and a vertical (top–bottom) axis.

Among the abovementioned investigations, it is noteworthy that a recent study (Teghil et al., 2021) explored if mental representations of autobiographic memories (AMs) unfold along a sagittal timeline. Autobiographical memory, the capacity to recall personal events of one’s life (Baddeley, 1990; Rubin, 1986), can be roughly classified into an episodic and a semantic component (Tulving, 1972, 1985). The episodic autobiographic memory (EAM) involves the recollection of past events with specific spatiotemporal details, enabling people to encode these personal events according to where and when they happened (Corballis, 2019). The semantic autobiographic memory (SAM), on the other hand, stores general and factual knowledge related to oneself (e.g., names of one’s primary school teachers, personalities of one’s best friends) without definite temporal or spatial information. Teghil et al. (2021) devised a spatial compatibility task in which native speakers of Italian were presented with linguistic labels that depicted events of their own lives (EAMs) and individual factual knowledge (SAMs) at different life periods. Participants had to judge the relative chronological order of the stimuli (i.e., if a linguistic label preceded or followed the previous one in chronological order) by pressing one of the two direction keys on a standard keyboard. In the compatible condition, the down arrow key was designated as “back/past” and the up arrow key as “front/future”, whereas in the incompatible condition this key assignment was reversed (down arrow = “back/future” and up arrow = “front/past”). Results showed that the participants were significantly faster when processing the order of EAMs in the compatible condition than in the incompatible condition. This is taken as evidence that representations of AMs (especially EAMs) develop along a sagittal back-to-front MTL. In light of the experimental results, Teghil et al. (2021) proposed that the back-to-front linear representations of AMs could actually be grounded in our sensorimotor experience related to moving (e.g., walking/running) through space. When walking and running, a person generally moves from back to front. Consistent with this directionality of locomotion, the recollection of personal events set in the past or in the future is respectively associated with the back or the front space, which constitutes a sagittal MTL.

Aside from the universal existence of the locomotor experience, there is another oft-cited origin of the sagittal MTL that might have escaped notice by Teghil et al. (2021). A growing body of neuroscientific and behavior experiments in the past decades has established that patterns in metaphorical language can profoundly modulate how people access front/back spatial representations to think about time (Gu et al., 2019; Miles et al., 2010; Torralbo et al., 2006; Ulrich et al., 2012; W. Yang & Sun, 2016a, 2016b; W. Yang et al., 2022). In many natural languages, spatiotemporal metaphors for temporal sequences indicate that passage of time slides along a sagittal line: the future is located *ahead of* and the past *behind* us (Haspelmath, 1997). This linguistic metaphoric mapping between time and space is commensurate with the egocentric sensorimotor experience of daily walking and running. However, despite the ubiquity of past/back and future/front associations in linguistic construals, directional variations do exist in the way specific languages map time flow onto the sagittal axis. Typical examples include, but are not limited to, Aymara, Mandarin and Vietnamese, which depict the future and the past as being respectively behind and in front of the body (Núñez & Sweetser, 2006; Starr & Srinivasan, 2021). The metaphoric patterns of these particular languages leave open an interesting question as to whether the representation of AMs along a back-to-front timeline is a universal phenomenon consistent across different linguistic communities. Therefore, a conceptual replication and novel extension of Teghil et al.’s (2021) research is crucial for addressing this issue. With respect to speakers whose native languages conceptualize elapsing time in an orientation (i.e., front-to-back) opposite to that of the sensorimotor experience, we envisage two possibilities concerning the directionalities of their AM sequences along a sagittal timeline:

1. The representation of AM proceeds along a back-to-front timeline that aligns with the sensorimotor experience such as walking and running.
2. The representation of AM develops along a front-to-back timeline that accords with spatiotemporal metaphoric mappings in these particular languages.

The present study focused on Mandarin, which predominantly employs front/past and back/future metaphors to describe time (H. Li, 2017; Wu, 2021; Y. Yang et al., 2023). For instance, while “*qián tiān*” refers to “the day *before* yesterday”, “*hòu tiān*” refers to “the day *after* tomorrow”. We conducted an experiment that recruited native speakers of Mandarin as participants to test the two hypotheses formulated above. The experimental design, adapted from Teghil et al.’s (2021) spatial compatibility paradigm, included a two-alternative forced-choice reaction-time (RT) task that asked participants to categorize personal events retrieved from their AMs as past- or future-related via distinct key arrangements corresponding to a congruent (i.e., along a back-to-front MTL) and an incongruent setting (i.e., along a front-to-back MTL) respectively. We sought to expand the scope of Teghil et al.’s (2021) research by clarifying which source (either sensorimotor experience or linguistic conventions) could reliably contribute to the sagittal MTL underlying AM organizations on the condition that the two critical factors contradict each other in spatial orientations.

## 2. Experiment

### 2.1. Participants

Fifty-two native speakers of Mandarin (30 females, *M*age = 31.08, *SD*age = 1.41, Range: 29–33) from P. R. China took part in this study in exchange for payment. To calculate the sample size through G*Power 3.1.9.6 (Faul et al., 2007), we assumed a medium-sized effect ($\eta_{p}^{2}$ = 0.25), an alpha level of 0.05, and a test power of 0.9, which yielded a recommended sample size of 44 participants. We thus slightly oversampled, factoring in certain attrition. All participants reported being right-handed and had normal or corrected-to-normal vision. They had already obtained a degree in tertiary education, thus having reached a high level of literacy in their native language.

### 2.2. Materials

One day prior to the spatial congruency RT task, we collected participants’ memories using an autobiographical memory fluency task adapted from Teghil et al.’s (2021) study. For each of six life periods (i.e., 6–10, 11–15, 16–20, 21–25, >26 years excluding last year, last year), the experimenters required that participants report as many personal events (EAMs) and names of friends, teachers, schoolmates or colleagues (SAMs) that corresponded to those periods. The participants were asked to generate a meaningful and personalized label for specific events or facts, which allowed them to explicitly identify these EAMs or SAMs. They were also instructed to report only events that were vivid and occurred at a specific time and place and to provide names of persons that were not associated with more than one life period. For each combination of period and category (i.e., EAM or SAM), 90 s were given. The participants reported, on average, a total number of 38.93 (*SD* = 8.64) EAMs and 59.88 (*SD* = 14.02) SAMs.

The first two items and the last two items for each period and category were used in the subsequent RT task. The criteria for item distribution in experimental setups were that either the first two items or the last two would appear in one trial, which guaranteed that each item unambiguously followed or preceded the other. Each participant completed four testing blocks, each consisting of 12 trials: an EAM congruent block, an EAM incongruent block, a SAM congruent block and a SAM incongruent block. The block order was counterbalanced across participants. Each label as referenced above would appear twice, respectively in the congruent and incongruent condition. For instance, an EAM label was included once in the EAM congruent block and once in the EAM incongruent block. Likewise, a SAM label would occur in the SAM congruent and SAM incongruent block respectively. The order of the trials was randomized within each block. Each block started with 2 additional practice trials, and the items used in the practice trials were not used subsequently in the formal testing trials.

### 2.3. Procedures

Right before the administration of the spatial congruency RT task, experimenters refreshed participants’ memories of the meaning of each label to minimize any potential misapprehension about the link between labels and memories.

**Figure 1:**
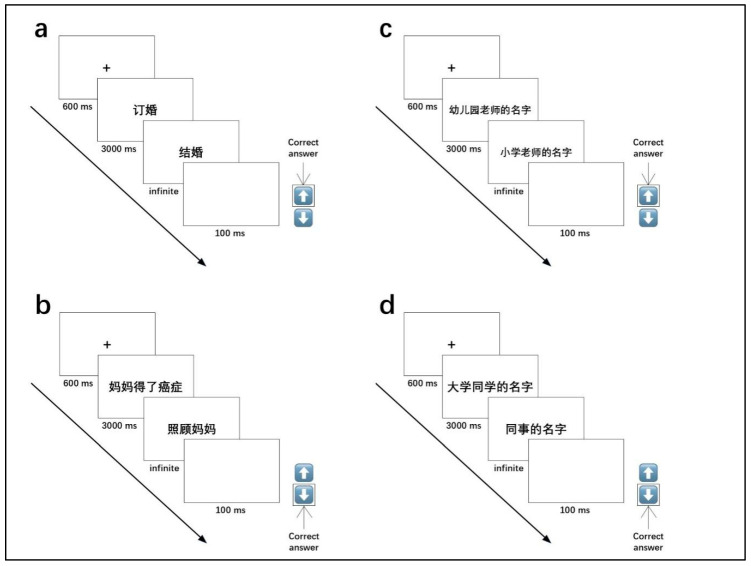
(**a**) An example of the EAM congruent condition. The English equivalents for the Chinese characters in the two stimuli in sequence are “engagement” and “marriage”. (**b**) An example of the EAM incongruent condition. The English equivalents for the Chinese characters in the two stimuli in sequence are “mom diagnosed with cancer” and “looking after mom”. (**c**) An example of the SAM congruent condition. The English equivalents for the Chinese characters in the two stimuli in sequence are “name of a kindergarten teacher” and “name of a primary school teacher”. (**d**) An example of the SAM incongruent condition. The English equivalents for the Chinese characters in the two stimuli in sequence are “name of a university classmate” and “name of a colleague”.

All participants were tested individually in a quiet room, using the same laptop computer with a 15.6-inch LED screen (32 bits, 1400 × 900 pixels, 8-ms refresh rate). On each trial, a red fixation cross was presented in the center of the screen for 600 ms. Then a label stimulus of the autobiographical events or facts selected from the fluency task production appeared in the center of the screen for 3000 ms, followed by another label stimulus that would remain on the screen until the participants made their judgments. In the congruent condition, the participants were instructed to decide whether the second stimulus preceded or followed the first stimulus in chronological order by pressing the “down” arrow and the “up” arrow respectively on the keyboard. On the contrary, in the incongruent condition, the participants were asked to press the “down” arrow if the second stimulus followed the previous stimulus and the “up” arrow if it preceded the last one chronologically. They needed to respond as quickly and accurately as possible. Upon entry of a response, a blank screen of 100 ms replaced the stimulus and a new trial began. An example of the trials and response keys was shown in Figure 1. RTs and accuracy were collected.

## 3. Results

Results from seven participants whose overall accuracy rate was lower than 85% were excluded from the dataset. The accuracy rate was determined based on our previous studies (e.g., W. Yang & Sun, 2016a; W. Yang et al., 2022), which adopted an RT paradigm to explore crosslinguistic differences in MTL. Trials that recorded a response latency more than 3 *SD* away from each participant’s mean respectively on the four testing blocks (8.56%) and trials on which the participants made errors (5.46%) were omitted from the RT analyses. The accuracy rate (91.07%, 91.21%, 92.02% and 91.46%) did not differ significantly [*F* < 1] across the four testing blocks (i.e., EAM congruent, EAM incongruent, SAM congruent, SAM incongruent) respectively.

**Figure 2:**
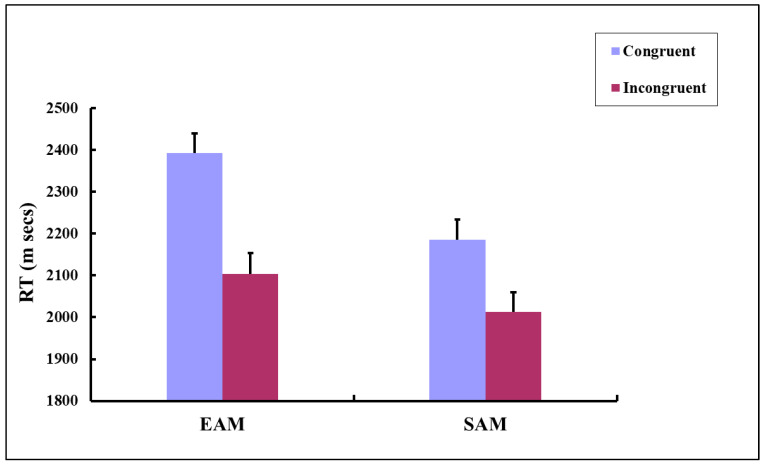
Mean RTs for stimuli of different categories in different conditions. Error bars indicate standard errors of the mean.

The remaining response data were submitted to a by-participants 2 × 2 [Category (EAM vs. SAM) × Condition (congruent vs. incongruent)] repeated measures ANOVA. The results revealed a significant main effect of Category: *F* (1, 44) = 18.25, *p* < 0.001, $\eta_{p}^{2}$ = 0.293, and of Condition: *F* (1, 44) = 15.46, *p* < 0.001, $\eta_{p}^{2}$ = 0.260. A nonsignificant Category × Condition interaction was observed: *F* (1, 44) = 1.84, *p* = 0.182. Planned paired *t*-tests showed that the participants responded to incongruent stimuli significantly faster than to congruent stimuli, irrespective of the category type [EAM congruent vs EAM incongruent: *t* (44) = 4.08, *p* < 0.001, *d* = 0.608; SAM congruent vs SAM incongruent: *t* (44) = 2.32, *p* < 0.05, *d* = 0.346]. The results also indicated that the overall RTs were longer for EAM than for SAM: *t* (44) = 4.27, *p* < 0.001, *d* = 0.637. Figure 2 plots the by-participants mean RTs in the spatial congruency task.

## 4. Discussion

The present study testified whether Mandarin speakers accommodate a sagittal back-to-front or a front-to-back MTL to represent AMs. The purpose of using a spatial compatibility RT design in the experiment is to examine whether the participants responded faster to conditions that presented space-time mappings commensurate with those that inherently exist in their cognition. We assume that when the space-time associations generated by the experimental setup conflict with either the sensorily general (i.e., back-to-front) or linguistically specific (i.e., front-to-back) mappings suggested by their conceptual representations, participants would suffer from interference and their response would slow. To be specific, if Mandarin speakers automatically map past AM components onto the back side of space (and future AM components onto the front), then their performance should be disrupted or slowed down if they are asked instead to map past AM components onto the front side of space (and future AM components onto the back). On the contrary, if Mandarin speakers automatically project the past to the front (and the future to the back), then their performance should be interfered or slowed down if they are asked instead to project past to the back (and the future to the front). The experimental results unequivocally match one of the aforementioned predictions. RTs in the incongruent condition were significantly shorter than those in the congruent condition for both EAM and SAM, which manifests that Mandarin speakers conceive of AM progression as oriented from front to back.

Given the findings of the current study, one of the most cogent explanations for the creation of a sagittal front-to-back MTL could be metaphoric language. As outlined earlier in this paper, Mandarin systematically uses front/past and back/future metaphors to delineate time. Note that backward metaphors coexist with forward expressions (i.e., front/future and back/past) in Mandarin to designate the sagittal directionality of time flow. However, Mandarin front-to-back metaphors have been documented to be the invariant and dominant space-time mappings over their contextually governed back-to-front spatiotemporal construals in experimental studies (Y. Yang et al., 2023). This discovery was underpinned by corpus and dictionary search, which verified that less than 15% of spatial metaphors for time along the sagittal axis in Mandarin were front/future and back/past terms (H. Li, 2017; Wu, 2021). In comparison with overwhelming front/past and back/future metaphoric patterns, the frequency and power of front/future and back/past expressions were therefore too trivial to construct a corresponding sagittal timeline in Mandarin speakers’ internal conceptualization of AMs.

Our discoveries also give rise to an important question: Why do spatiotemporal metaphors rather than sensorimotor experience enact a critical role in shaping the sagittal MTL underlying AM representations provided that the two forces progress in reversed directions? It is acknowledged that sensorimotor experience and conceptual experience (e.g., linguistic metaphors) are both possible sources of space-time mappings along the sagittal axis (Jiang et al., 2023). Nevertheless, the two types of experience involve distinct levels of perception processes. Sensorimotor experience, e.g., walking and running, is an intra-conceptual and low-level embodied simulation, which posits that perceptual processing entails the simulation or neural reactivation of associated bodily states derived from prior experiences (Barsalou, 1999, 2008). On the other hand, conceptual experience like spatiotemporal metaphors is a high-level process, in which the inter-conceptual mechanism maps content and structure between source concept and dissimilar target concept (Landau et al., 2010). Previous research has demonstrated that low-level sensorimotor experience would be inhibited by high-level processes such as language and culture if the two forces compete with each other for the same cognitive channel/resources (Awh et al., 2004; Ding et al., 2020; Jiang et al., 2023). Therefore, the specific spatiotemporal metaphors were spontaneously activated and the universal sensorimotor experience automatically restrained or even eliminated to compete for the directionality of MTL, which brought about a front-to-back timeline in the temporal organization of Mandarin speakers’ AMs.

Herein we suggest an additional variable, namely cultural attitudes toward time, that may potentially modulate the sagittal front-to-back representation for Mandarin speakers’ AMs. Accumulating evidence has revealed that whether one attributes more importance to the past or the future in one’s culture could condition people’s spatial conceptions of time (de la Fuente et al., 2014; Valenzuela et al., 2020). Influenced by the common belief in Confucianism, Chinese as well as some other oriental cultures encourage more positive thinking of past times and old generations than of the future and young populations (Chung & Lin, 2012). Mandarin speakers were accordingly shown to be past-focused and exhibit a salient propensity to conceptualize the past as ahead of and the future as behind their bodies in experimental inquiries (Gu et al., 2019; H. Li & Cao, 2017). Similar interpretations can be found in Guo et al. (2012), which observed that Mandarin speakers attached greater monetary value to past events than to future events, indicating a front-to-back bias for representations of temporal relations. Insofar as Mandarin speakers’ performances in the present study are concerned, we hypothesize that linguistic metaphors, together with cultural attitudes toward time, may presumably form a dual power mechanism that overrides the effect of sensorimotor experience related to walking/running but reinforces the front/past and back/future metaphoric mapping between time and space for AMs in Mandarin speakers. In summary, cultural attitudes toward time may serve as an implicit modulator that provides alternative and plausible accounts of the origin underlying the sagittal MTL for AM representations. Justifying the reliability of this speculative interpretation, though outside the parameters of the present study, could be a promising line for further research.

Finally, an interesting issue that emerged from this study is yet to be addressed: Why were the participants’ overall RTs longer for EAM than for SAM? Several empirical works (e.g., Karylowski & Mrozinski, 2017) have indicated that EAM, as compared with SAM, could facilitate faster retrieval and processing when participants made self-judgments based on retrieved autobiographical content. Our experimental results appear to conflict with these prior findings. Nonetheless, some other existing literature has proposed that EAM can be retrieved and processed faster than SAM (e.g., J. Li & Guo, 2009; Liang, 2021). The retrieval of EAM entails an explicit reactivation of specific spatial and temporal contexts in the past, which could increase the cognitive endeavor to search for relevant clues. This procedure, vulnerable to interferences from various factors, is assumed to be relatively complex and slow. The retrieval of SAM, on the contrary, is presumed to be a relatively fast and automatic process. It involves a direct and quick index of factual knowledge without consciously recalling definite spatial or temporal elements, thereby reducing the cognitive load associated with information filtering. These positions or experimental findings, taken together, suggest that the dispute pertinent to response latencies underlying EAM versus SAM retrieval remains unresolved, which merits further consideration by subsequent studies. What is clear thus far, however, is that the critical statistical comparison implemented by the current investigation lies in RTs between the congruent and incongruent conditions within the EAM and SAM categories respectively. In other words, the controversies instantiated above regarding EAM versus SAM retrieval do not undermine the spatiotemporally compatible effects observed within each memory category for the present study.

## 5. Conclusions

The present study corroborates the psychological reality of a sagittal MTL that governs the temporal sequence of AM processes. Moreover, we clarify and extend the work of Teghil et al. (2021), demonstrating that the back-to-front orientation of the MTL, grounded in sensorimotor experiences such as walking or running, may not be a universal phenomenon across linguistic communities. The preliminary evidence procured from Mandarin speakers adds to the existing literature by highlighting the robust role of spatiotemporal metaphors in shaping a front-to-back axis for temporal cognition in general (e.g., Gu et al., 2019; Walker et al., 2014, 2017) and for the temporal organization of AMs in particular. Actually there are some other languages (e.g., Aymara and Vietnamese) that encode the passage of time along a front/past and back/future linear layout. Follow-up investigations may recruit speakers of these native languages as participants to further validate the predictive power of linguistic metaphors over that of sensorimotor experience in constructing a sagittal MTL for Ams, based on the premise that the two factors contradict each other in spatial directions.

## 6. Limitations

The present study has a potential limitation, given that we manipulated an indirect rather than a direct crosslinguistic comparison in the core experimental design. This study did not simultaneously recruit two linguistic groups, i.e., Mandarin and Italian speakers, in the same experiment. Instead, we conceptually compared the Mandarin data to that of Teghil et al. (2021). Some confounding variables (e.g., differences in recruitment, exclusion criteria for data, experimental apparatus and context) were consequently outside of the experimental control. These factors, other than metaphorical language, may also be associated with the apparent discrepancies between Mandarin and Italian speakers. Therefore, the conclusions drawn and the underlying explanations elaborated in the paper, although highly plausible, should be treated with caution and call for further examination.

## Data Availability

The data presented in this study are available on request from the corresponding author. The data are not publicly available due to privacy concerns.
